# Molecular data suggest population expansion and high level of gene flow in the Plain Tiger (*Danaus chrysippus*; Nymphalidae: Danainae)

**DOI:** 10.1080/23802359.2018.1483751

**Published:** 2018-06-26

**Authors:** Vinaya Kumar Singh, P. C. Joshi, Bheem Dutt Joshi

**Affiliations:** a Department of Zoology and Environmental Science, Gurukula Kangri Vishwavidayalaya, Haridwar, India;; b Wildlife Institute of India, Dehradun, India;; c Zoological Survey of India, Kolkata, Inida

**Keywords:** Genetic diversity, stepping stone, incomplete lineage sorting, butterfly, Western Himalaya

## Abstract

In the present study, we sequenced the individuals of the *Danaus chrysippus* from the different altitudinal ranges in the western Himalayan state of Uttarakhand, India and compared with other global published data across its distribution range using the cytochrome oxidase c subunit-I (COI). Among the sequences generated in this study, we observed total six haplotypes with intra-species sequence divergence of 0.001–0.009. Whereas the combined data generated a total of 24 haplotypes. The genetic diversity and neutrality test indices suggested overall population expansion of the species. This has also been supported by the MJ Network as it shows the star-like topology and formation of one core haplotype with maximum frequency distribution to the multiple locations. Whereas, the phylogenetic tree shows mixing of the haplotype from the different locations to the same clade. Haplotype arrangement in both phylogenetic tree and MJ Network is not clustered as per their geographic affinity, which suggests incomplete lineage shorting and recent population expansion or colonizations to the new area.

## Introduction

Genetic-based studies are important to understand the genetic diversity, diversification pattern and phylogeography of the species (Zhang and Jiang 2006; Bärmann et al. 2013). Butterfly are good indicator species to detect any oscillation in regional climatic condition and expand or contract their distribution range accordingly (Thomas 2005). There are limited or no genetic studies available from the western Himalaya on butterflies. *Danaus chrysippus*, a butterfly species distributed in the different altitudinal gradients in this region. The *D. chrysippus*, commonly known as the plain tiger or African monarch, believed to be first recorded butterfly species in Egypt and used in the art (Larsen, 1977, 1984, 1994). It belongs to order Lepidoptera, family Nymphalidae and Danainae (milkweed-butterflies) subfamily of the brush-footed butterfly. This species extensivelly studied in much detailed using molecular markers than other members of its subfamily found in Africa, southern Europe and Asia, but DNA based studies are lacked from India except few sequences available of cytochrome oxidase *c* oxidase I (COI) gene from southern part of India. The individuals of this species migrate in large numbers to the south in winters and north in summers and do not form discrete populations may be because of the adult’ individuals are long-lived and have a migration capability in search of nectar, pyrrolizidine alkaloid sources, mates and food-plants (Boppré 1990).

The geographical distributions of this species have subsequently been refined and essentially confirmed (Rothschild et al. 1975; Smith et al. 1998; Lushai et al. 2003 a, b, c). Several studies on butterflies provided convincing evidence of climate change on range distribution, extinction risk (Parmesan et al.^1999^; McLaughlin et al. 2002) and predictable responses to change in the environmental conditions (Scriber et al. 2002, Crozier 2004).

The present study is aimed to characterize the genetic variability of individuals of *D. chrysippus* collected from lowland and high altitude in the state of Uttarakhand, India using the COI gene. As this marker has been broadly screened in a variety of species to document the genetic diversity, phylogeography and population genetics analysis (Hebert et al. 2003; Negi et al. 2016). Further, we compared the sequences of other geographic locations throughout its distribution to understand the haplotype distribution, phylogeography and overall demographic pattern.

## Materials and methods

The samples were collected from selected regions of Garhwal and Kumaun regions in Uttarakhand, India. The samples were collected from six sites viz., RajaJi National Park (Altitude: 300m, Coordinate 30° 05′ 58.76′′ N, 77° 58′ 29.82′′ E), Kaladhungi (Altitude: 610 m, Coordinate 29°16′48.45′′ N,79°21′00′′ E), Jeliokot (Altitude: 1370m Coordinate 29° 20′ 34.17′′N,79° 29′ 01.45′′E), Kailakhan (Altitude:1820m, Coordinate 29° 22′27.03′′N,79° 28′ 33.92′′E), Snow View (Altitude: 2252 m, Coordinate 29° 23′43.03′′N,79° 27′ 13.01′′E) and China peak/Naina peak (Altitude: 2611 m Coordinate 29° 24′33.06′′N, 79° 26′ 23.12′′E). Butterflies were collected using the sweeping net method as per Gadagkar et al. (1990), then entire samples were stored in insect box (Herbarium). Each individual was identified through observing the wing shape and color pattern description available in keys/identification guides (Antram 1924; Peile 1937; Gunthilagaraj et al. 1998; Kunte 2000; Rangnekar 2007). Butterflies were spread and preserved according to the standard entomological methods. One or more legs and thorax part were removed and kept in ethanol for long time storage. Total 30 samples of butterflies were used for the DNA extraction. Genomic DNA was obtained from legs and thorax tissues using a HiPurATM Insect DNA Purification Kit following the protocol of respective kit (Hi-Media). A fragment 658 bp was amplified using universal DNA primers of mitochondrial COI (LCO1490-F-5′-GGTCAACAAATCATAAAGATATTGG3′ and HCO2198-R-5′-AAACTTCAGGGTGAC CAA AAAATCA-3′) gene (Folmer et al. 1994). Polymerase chain reaction (PCR) amplification was carried out using 10 µl of PCR master mix with 1.5X PCR buffer, 2.5 mM MgCl2, 200µM dNTP, 0.4µM of each primer, 0.5U Dream Taq polymerase (Thermo Fisher Scientific) and 40 ng of genomic DNA. The thermal cycling parameters of PCR included initial denaturation at 95 °C for 3 min and at 95 °C for 40 s annealing at 45 °C for 50 s and 72 °C for 50 s and one cycle of a final extension for 10 min at 72 °C. PCR amplification was checked by loading 2 µl of the reaction mixture on a 2% (w/v) agarose gel. The amplified PCR products were then processed for cycle sequencing PCR with their respective forward primers, using a master mixture which was prepared following the method of Applied Biosystems. These products were then subjected to DNA sequencing on ABI 3550 genetic analyser.

## Data analysis

The quality of the sequences were checked on Sequencher 4.7 (Gene Codes, USA). Multiple sequence alignments were done using the Clustal W in Bioedit version 7.0.9.0 (Hall 1999). The generated sequences were compared with available sequences of COI gene for *D. chrysippus* in NCBI database and confirmed. Sequences were also retrieved from the NCBI (*n* = 54) of this species which includes a total of 84 (obtained from the present study + NCBI). We categorized them into 10 populations (major zones) of *D. chrysippus* from different locations. Additionally, one individual of the congeneric *Danaus petilia* was included as an out-group. The population zones (B and D) included Thailand, Myanmar, Vietnam, Indonesia and Australia were represented by seven to ten individuals, while eight population zones (A, C, E, F, G, H, I and J) which included (Taiwan, China, Southern India, Pakistan, Philippines, Western India, Kenya, Uganda, Togo, Republic of Korea, Central African, Cameroon, NBR Mukuruthi National, USA, and Northern India) have four to six individuals from each zone.

The phylogenetic tree was constructed using a Bayesian method using BEAST software package (Drummond et al. 2012). Character attributes were identified manually to find the specific nucleotide in the data sets in Bioedit and MEGA 7. The distribution of genetic variation in *D. chrysippus* samples was analysed using Arlequin 3.11 (Excoffier et al. 2005). Population zones with less than five individuals were excluded from this analyses and the nucleotide (*π*) and haplotype (*h*) diversity Tajima’s *D* and Fu’s *F*s were calculated using DnaSP (Rozas et al. 2003) and Arliquin (Excoffier et al. 2005).

## Results

**Figure 1.**
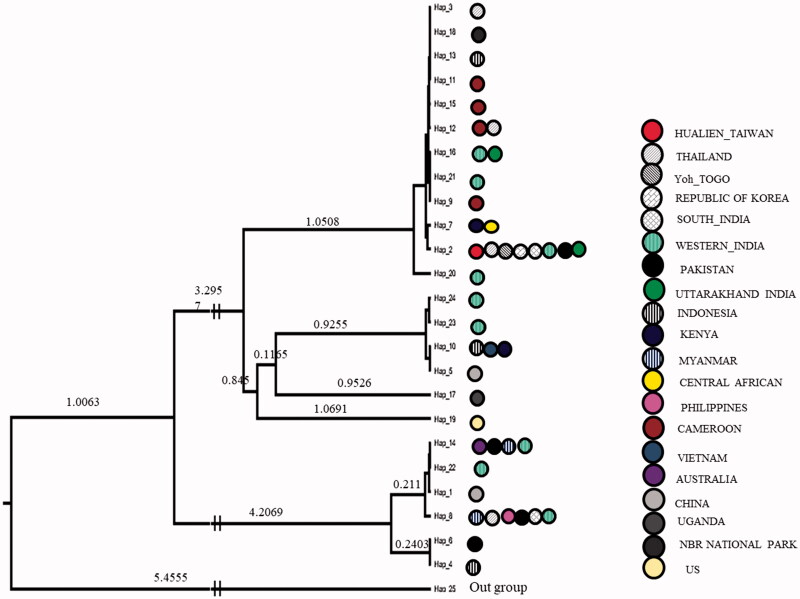
Bayesian phylogenetic tree constructed in BEAST software and *Danaus petilia* as the outgroup.

**Figure 2.**
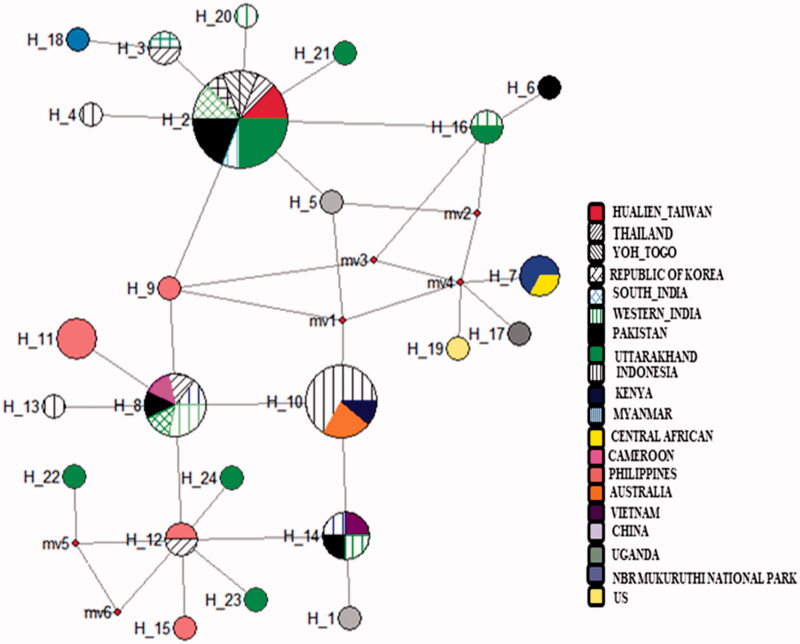
MJ – a network of the 24 D. chrysippus haplotypes reconstructed in NETWORK. Each circle represents haplotypes, and the diameter of the circle is approximately proportional to the number of samples present in the haplotypes. Colour represents the geographic locations of the haplotype.

DNA from all the thirty samples was extracted successfully and generated good quality sequences of COI gene. These 30 sequences generated from Uttarakhand had a total of eight variable sites and observed six haplotypes with the sequence divergences ranges 0.001–0.009. For the comparative analysis, a total of 54 sequences of *D. chysippus* were retrieved from the NCBI and combined with our seqeuences (*n* = 30), which generated total 31 variable sites and have the intra-species sequence divergence range 0.001–0.018. The nucleotide compositions in combined sequences were 39.8% T, 15.6% C, 31.1% A and 13.5% G. All the combined sequences generated a total of 24 haplotypes. The nucleotide and haplotype diversity of all sequence generated in the present study were 0.02336 and 0.086, respectively (Table 1). Whereas, Tajima’s *D* and Fu’s *Fs* value were 1.80377 and −1.178, respectively, and found statistically significant at (*P* < .02) (Table 1). Further, all the sequences grouped into the 10 zones based on their geographic affinity in which nucleotide and haplotype diversity in each of these zones were 0.00229–0.01122 and 0.378–0.933, respectively. Tajima’s *D* and Fu’s *Fs* values were found negative in all populations but statistically non-significant (Table 1). The MJ network for the sequence generated in the present study was not generated separately but combined with all samples data, which spanned with total of 24 haplotypes. Among these observed six haplotypes in this study, four haplotypes (H21, H22, H23 and H24) form a separate clustered irrespective to their geographic affinity, but not shared with other haplotypes and constituted as new haplotypes, whereas haplotype 2 were the part of the core haplotype and haplotype 16 shared with other sequences from the different parts (Figure 2). The overall MJ network characterized by the star-like topology. In MJ Network haplotype 2, 8, 10, 12 and 14 have a maximum frequency of samples originated from 4 to 8 locations. Haplotype two was highly distributed among the locations and throughout its distribution range. Phylogenetic tree using Bayesian approach, all the samples broadly form three major clustered. The sequences generated in this study were clustered in these three clades, and similarly, all the samples show mixed arrangements (Figure 1).

**Table 1.** Mitochondrial diversity indices and neutrality tests values observed using COI gene of mtDNA genome in *Danaus chrysippus* (butterfly) from the present study and other published study.

S.No.	Loc.	*N*	*S*	Nh	Nucleotide diversity (*π*)	Haplotype diversity (h)	Tajima’s *D*	Fu’s *F*s	References
**1**	Chaina, Taiwan	05	05	3	0.00445	0.70	−0.56199	0.804	–
**2**	Thailand, Vietnam, Myanmar	07	05	5	0.00463	0.90	0.59446	−1.262	–
**3**	Southen India	04	03	3	0.00297	0.83	−0.75445	−0.288	–
**4**	Indonesia, Australia	10	05	3	0.00229	0.37	−1.38818	0.762	–
**5**	Pakistan	06	06	4	0.00475	0.80	−0.49605	−0.168	–
**6**	Philippines	06	05	4	0.00422	0.80	−0.14427	−0.382	–
**7**	Western India	06	07	5	0.00574	0.93	−0.37783	−1.372	–
**8**	Kenya, Uganda	04	11	3	0.01122	0.83	−0.55827	1.702	–
**9**	Togo, Korea, African, USA	06	18	5	0.01320	0.93	−0.95976	0.142	–
**10**	Present study	30	51	7	0.02336	0.86	−1.80377*	1.178*	–
	Overall	84	75	25	0.00933	0.90	−2.43622*	−9.497*	–
*Comparison of diversity indices with other species*									
	Species								
1	*D.chrysippus*	–	–		**0.02336**	**0.86**	–	–	Current study
2	*Lopinga achine*	–	–		0.00000	0.84	–	–	Ullasa et al. (2012)
3	*Aglais urticae*	–	–		0.01000	0.97	–	–	
4	*Melitaea cinxia*	–	–		0.02000	0.95	–	–	Wahlberg and Saccheri (2007)
5	*Mycalesis orseis*	–	–		0.00200	0.75	–	–	Suzan et al. (2007)
6	*Sitodiplosismosellana*	–	–		0.01100	0.87	–	–	Hong et al. (2006)
7	*Danaus plexippus*	–	–		0.0007	0.46	–	–	Edward et al. (2017)

N = number of samples, S = segregation sites, Nh = number of haplotype diversity.

*Statically significant.

## Discussion

Present study highlighted the genetic diversity along the altitudinal gradient of *D. chrysippus* from 300 to 2611 m in Garhwal and Kumaun regions of Uttarakhand. Further, comparative analysis, within country range suggest that sequences have low sequence divergence 0.001–0.009. Whereas with sequences of the other part of its distribution range falls within the range of intra-species sequence divergence (Table 2; 0.001–0.018) and a similar range of sequences divergence reported in the other species (*Magicicada septendecim* is 0.05 and *Drosophila silvestris* is 0.3). Low sequence divergence among distant geographic location indicates the high geneflow and recent population expansion/colonization into these areas. Further, the nucleotide and haplotype diversity also supports the population expansion observed in this study because the species that are highly vagile and migratory have less chance to subdivide into nearly isolated demes or populations. These negative and statistically significant values of neutrality test also support the population expansion (Pierce et al. 2014; Edward et al. 2017). However, the sample size is low to draw this conclusion as in the some of sites, few sequences were included, which may be one of the reasons for the low genetic diversity. However, this is performed with the overall samples analysed from the each of zones, where we observed the same pattern of the population expansion and haplotype sharing from its distribution range. The long-lived adults of this species also migrate to long distance in search of nectar, pyrrolizidine alkaloid sources (Boppre 1984), mates and food-plants and use different habitat as stepping stones. These findings suggest that these species have a strong sign of population expansion and continuous geneflow among the locations. Whereas, in combined data, total 8 haplotypes were shared out of 24 haplotypes between the locations and that of haplotype no. 2 were distributed in maximum frequency across the different sampling locations. In the comparative diversity indices of the different zones, high nucleotide diversity was observed in zone *G* (0.933) and lowest in zone *D* (0.378), respectively, whereas highest haplotype diversity in zone *H* (0.02336) and lowest in zone *D* (0.00229), respectively (Table 1). Whereas these nucleotide and haplotype diversity in different zone found comparable to only a few species which ranges from 0.00297 to 0.02336 and 0.378 to 0.933, with respect to other species (Table 1). Neutrality test, i.e. Tajima’s *D* and Fu’s *Fs* for the present study was found statistically significant, which is same as reported in *Danaus plexippus* (Edward et al. 2017). Further, in the MJ network, star-like topology was formed which support the values of the genetic diversity and neutrality test in support to population expansion (Ullasa et al. 2012, Suzan et al. 2007; Hong et al. 2006).

**Table 2.** Sequence divergence between the 10 populations of *D. chrysippus.*

Zone	A	B	I	C	D	E	H	F	G	J
Chaina, Taiwan **(A)**										
Thailand, Vietnam, Myanmar **(B)**	0.004									
Togo, Korea, African, USA **(I)**	0.006	0.007								
Southen India **(C)**	0.002	0.003	0.006							
Indonesia, Australia **(D)**	0.004	0.003	0.007	0.004						
Pakistan **(E)**	0.003	0.003	0.006	0.002	0.004					
Kenya, Uganda **(H)**	0.008	0.008	0.010	0.008	0.006	0.008				
Philippines **(F)**	0.005	0.003	0.007	0.004	0.004	0.004	0.009			
Western India **(G)**	0.003	0.003	0.006	0.003	0.003	0.003	0.008	0.004		
**Uttarakhand India (J)**	0.011	0.011	0.015	0.011	0.012	0.011	0.017	0.012	0.011	0.000

All these findings may be corroborated with the personal observation that we made during field, we suggest that this species has expanded their range in the high densities areas of human disturbance such as farms, gardens, wasteland and roadsides in the Western Himalayan region. But this may be a recent phenomenon, similarly, open grassland was found to have high species richness followed by forest patch, roadside plantation. These results may be due to the invasion of disturbed areas by generalized and widespread herb and shrub species such as Lantana camera etc. which act as a rich nectar source for butterflies (Kuladip et al. 2012). From the present study, it may be concluded that incomplete lineage shorting of COI gene may also lead to high haplotype sharing due to recent colonization.
